# Interaction between *HLA-B60* and *HLA-B27* as a Better Predictor of Ankylosing Spondylitis in a Taiwanese Population

**DOI:** 10.1371/journal.pone.0137189

**Published:** 2015-10-15

**Authors:** James Cheng-Chung Wei, Henry Wong Sung-Ching, Yu-Wen Hsu, Ya-Feng Wen, Wen-Chang Wang, Ruey-Hong Wong, Hsing-Fang Lu, Floris A. van Gaalen, Wei-Chiao Chang

**Affiliations:** 1 Division of Allergy, Immunology and Rheumatology, Department of Medicine, Chung Shan Medical University Hospital, Taichung, Taiwan; 2 Institute of Medicine, Chung Shan Medical University, Taichung, Taiwan; 3 Institute of Intergrative Medicine, China Medical University, Taichung, Taiwan; 4 Master Program for Clinical Pharmacogenomics and Pharmacoproteomics, School of Pharmacy, Taipei Medical University, Taipei, Taiwan; 5 Department of Clinical Pharmacy, School of Pharmacy, Taipei Medical University, Taipei, Taiwan; 6 The Ph.D. Program for Translational Medicine, College of Medical Science and Technology, Taipei Medical University and Academia Sinica, Taipei, Tiawan; 7 The Ph.D. Program for Translational Medicine, Taipei Medical University, Taipei, Taiwan; 8 Department of Public Health, Chung Shan Medical University, Taichung, Taiwan; 9 Department of Occupational Medicine, Chung Shan Medical University Hospital, Taichung, Taiwan; 10 Department of Pharmacy, Taipei Medical University-Shuang Ho Hospital, Taipei, Taiwan; 11 Department of Rheumatology, Leiden University Medical Center, Leiden, The Netherlands; 12 Department of Pharmacy, Taipei Medical University-Wanfang Hospital, Taipei, Taiwan; 13 Center for Biomarkers and Biotech Drugs, Kaohsiung Medical University, Kaohsiung, Taiwan; Kaohsiung Chang Gung Memorial Hospital, TAIWAN

## Abstract

**Objective:**

Ankylosing spondylitis (AS) is a form of chronic inflammatory spondyloarthritis (SpA) that causes pain and stiffness in spines or joints. Human leukocyte antigen B27 (HLA-B27) and B60 (HLA-B60) have been reported as major genetic risk factors of AS. In addition, rs13202464, located on major histocompatibility complex (MHC) region, showed high sensitivity (98.7%) and specificity (98.0%) for HLA-B27.

**Design:**

The aim of our study is to test whether the interaction between HLA-B60 and HLA-B27 (rs13202464) can serve as a better predictor of AS. We have genotyped HLA-B60 and rs13202464 among 471 patients with AS and 557 healthy subjects. Combined risk factors were investigated to test the biological interaction.

**Results:**

Our results indicated that the relative risk (RR) for HLA-B27+/HLA-B60− was 152 (95% CI 91 to 255) and it increased to 201 (95% CI 85 to 475) in HLA-B27+/HLA-B60+ patients (with HLA-B27−/HLA-B60− as reference). Combinational analysis of two risk factors (HLA-B27+/HLA-B60+) showed a relative excess risk due to interaction (RERI) of 46.79 (95% CI: -117.58 to 211.16), attributable proportion (AP) of 0.23 (95% CI: -0.41 to 0.88) and a synergy index (S) of 1.31 (95% CI: 0.56 to 3.04).

**Conclusion:**

In conclusion, genetic interaction analysis revealed that the interaction between HLA-B60 and HLA-B27 is a better marker for the risk of AS susceptibility in a Taiwanese population.

## Introduction

Ankylosing spondylitis (AS) is a chronic inflammatory disease leading to pain, stiffness and possible fusion of spinal segments. It is considered as a chronic, inflammatory disorder and affects sacroiliac joints, lumbar spine, and peripheral joints [1]. Progression of disease in AS patients often leads to limited of mobility, functional impairment and finally affects the patients’ well-being [2]. Prevalence of AS in men is higher than in women [3], while the pathological mechanisms of AS remain unclear [4].

A genome-wide association study (GWAS) conducted by The Australo-Anglo-American Spondyloarthritis Consortium (TASC) revealed the association of *HLA-B27*, *IL-23* and *IL-1* genes to AS [5]. *HLA-B27* gene is the best-known genetic susceptibility marker for AS, however, it only explains for 16% of the genetic variability in AS [6,7]. Associations between AS and the *HLA-B27* gene and *HLA-B60* gene have also been revealed [8]. In addition, Wei *et al*. showed that *HLA-B60* is a risk factor for *HLA-B27* negative patients [9]. In 2013, epistasis between *HLA-B27* and *HLA-B60* has been reported to associate with increased risk of AS in Caucasians, with a very high relative excess risk [10].

rs13202464, located on major histocompatibility complex (MHC) region, showed high sensitivity (98.7%) and specificity (98.0%) to for *HLA-B27* [11]. Another GWAS in Han populations also indicated that rs13202464 of *HLA-B* can represent the risk effects of *HLA-B27* in a Chinese population [6]. In this study, we investigated the correlation between *HLA-B27* and *HLA-B60* and the risk of AS. The association between *HLA-B27/HLA-B60* and disease severity of AS was also tested.

## Materials and Methods

### Subject recruitment

The patients with AS and the healthy subjects were from the Chung Shan Medical University Hospital. All of the participants recruited were ethnic Taiwanese. AS patients who met the New York AS diagnosis criteria were recruited to participate. Our study was approved by the institutional review boards of the Chung Shan Medical University Hospital in Taichung, Taiwan. Informed consent was obtained and be written before any data were collected from the subjects. Our study was approved by the institutional review boards of the Chung Shan Medical University Hospital in Taichung, Taiwan. Informed consent was obtained and be written before any data were collected from the subjects. This study has included the patients with age below 18 years old, with youngest subjects enrolled with age 17. These patients are considered adults by our Ethics Committee, and also the informed consent was obtained and be written before any data were collected from these subjects. The Bath AS Disease Activity Index (BASDAI), Bath AS Functional Index (BASFI), and Bath AS Global (BAS-G) which evaluate disease activity, physical function, and global well-being is collected by questionnaire. Modified Chinese versions of the BASDAI, BASFI, and BAS-G showed good intra-class correlations and Cronbach’s alpha values.

### DNA extraction and HLA Genotyping

DNA of blood cells were extracted by first treating them with 0.5% sodium dodecylsulfate lysis buffer and then protease K (1 mg/ml) to digest nuclear proteins for 4 h at 60°C. Total DNA was harvested using a Gentra (Qiagen, Valencia, CA) extraction kit followed by 70% alcohol precipitation. DNA purification from buffy coat was carried out by using the Gentra Puregene Blood Kit (Qiagen, Valencia, CA, USA). rs13202464 (HLA-B27) were genotyped by using the TaqMan® Allelic Discrimination Assay (Applied Biosystems, Foster City, CA). HLA-B60 positivity is identified by two separated SYBR Green real-time PCRs. A 96-well micro-plate with an ABI9700 Thermal Cycler (Applied Biosystems) is used to perform polymerase chain reaction (PCR). After PCR, fluorescence was detected and analyzed by StepOne software vers. 2.2.2 (Applied Biosystems) [12].

### Data analysis

Previous studies showed that genotypes of rs13202464 can tag to *HLA-B27*. Thus, we classified all subjects into *HLA-B27* positive (GG and AG genotype) or negative (AA genotype) by rs13202464 genotypes [11]. To examine the interaction effect between *HLA-B27* and *HLA-B60*, samples were categorized into four groups: *HLA-B27*+/*HLA-B60*+, *HLA-B27*+/*HLA-B60*-, *HLA-B27*-/*HLA-B60*+, *HLA-B27*-/*HLA-B60*- based on the genotyping results. The odds ratios (ORs) were calculated to assess the additivity of interaction effect, which use the sample of *HLA-B27*-/*HLA-B60*- as reference. The prevalence of AS in Taiwan is rare (0.167%) according to the definition of World Health Organization and this satisfies a rare disease assumption thereby replacing RRs with ORs. We used CaTS to calculate the power of association between HLA-B27/B60 to AS susceptibility [13].

Interaction is defined as a departure from additivity of effects. Three indices have been used to evaluate the biological interaction between *HLA-B27* and *HLA-B60*: (1) RERI: the relative excess risk due to interaction. (2) AP: the proportion of disease among those with two risk factors that is attributable to its interaction. (3) S: synergy index. RERI and AP should be zero and S should be 1 when no interaction was detected between two exposures. To obtain the parameter estimates needed for calculating these three measures, a logistic regression model was fitted [14]. The Statistical Package for the Social Sciences (SPSS) V. 20.0 (SPSS, Chicago, Illinois, USA) and R software (http://cran.r-project.org/), were used to analyse the data. Package *epiR* was used for biological interaction analysis. In all tests, p values less than 0.05 were considered significant.

## Results

As shown in Table 1, a total of 1028 subjects were recruited including 471 patients with AS, and 557 healthy subjects. The number of male in AS patients and normal subjects was 320 (67.9%) and 435 (78.0%). The mean of age in both groups were 39.0 years. *HLA-B27* and *HLA-B60* genotype data were collected from both patients and control subjects. Four hundred and thirty one (91.5%) AS patients and forty three (7.7%) control subjects were categorized as *HLA-B27*+. Besides, the number of *HLA-B60*+ was one hundred (21.3%) and seventy three (13.1%), respectively. The ORs of *HLA-B27* and *HLA-B60* to AS have been 120.80 (95% CI = 83.31 to 204.82) and 1.79 (95% CI = 1.29 to 2.49), respectively (Table 2).

**Table 1:** Basal characteristics of patients with ankylosing spondylitis (AS) and control subjects.

Characteristics	Patients with AS	Control subjects
Number of subjects	471	557
Gender: male (No (%))	320 (67.9%)	435 (78.0%)
Age (years)^a^	39.0 ± 11.3	39.0 ± 12.2
Range	17–82	17–77
*HLA-B27*(+)	431 (91.5%)	43 (7.7%)
*HLA-B60*(+)	100 (21.3%)	73 (13.1%)

^a^Mean ± SD. SD:standard deviation.

**Table 2:** HLA association with AS in Taiwanese population.

	AS no (%)	Controls no (%)	OR (95% CI)
*HLA-B27*+	431 (91.5%)	43 (7.7%)	120.80 (83.31–204.82)*
*HLA-B27*-	40 (8.5%)	514 (92.2%)	
All	471 (100%)	557 (100%)	
*HLA-B60*+	100 (21.3%)	73 (13.1%)	1.79 (1.29–2.49)*
*HLA-B60*-	371 (78.7%)	484 (87.1%)	
All	471 (100%)	557 (100%)	

*p<0.001. No, number of individuals.

**Fig 1:**
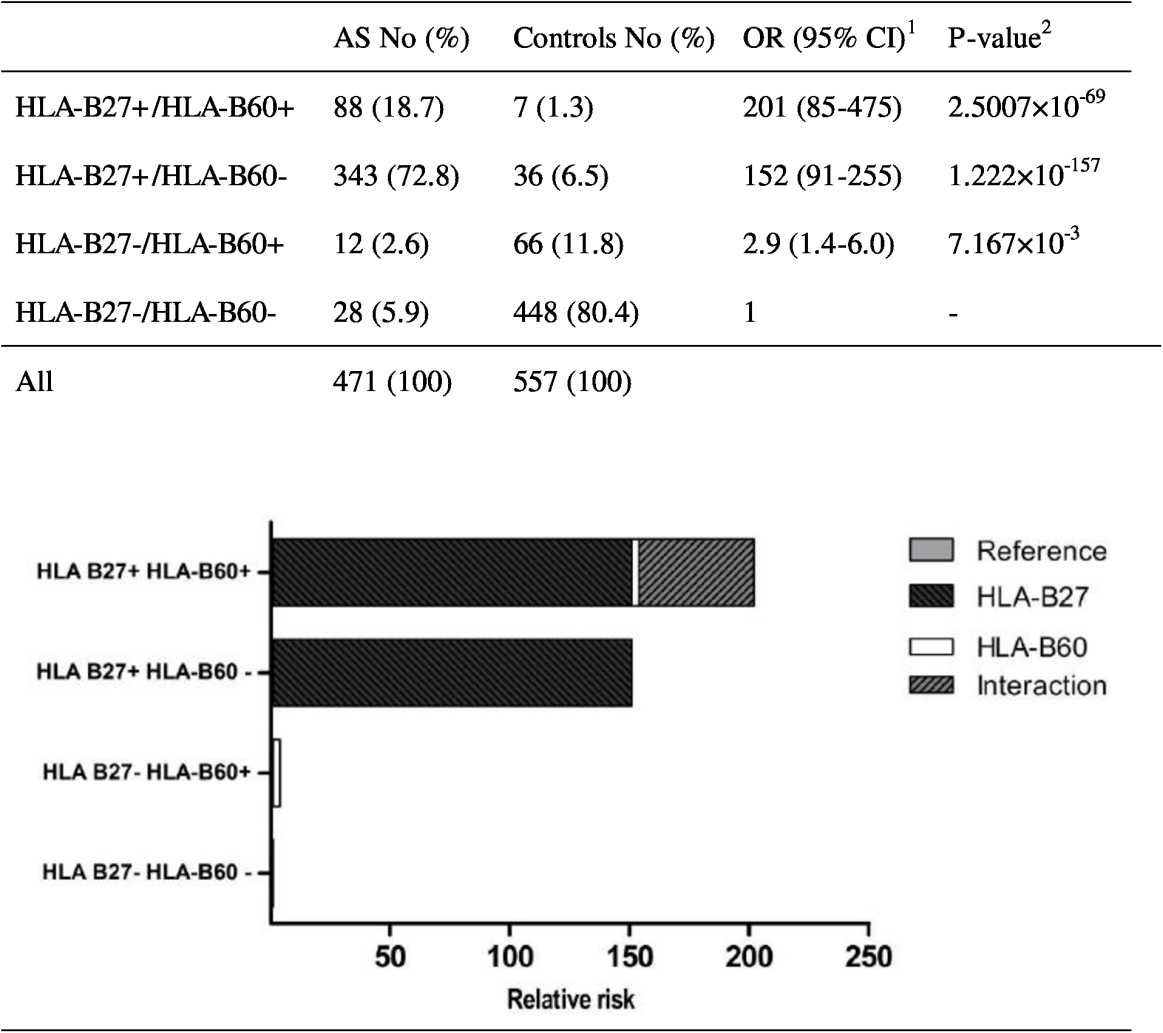
Interaction between HLA-B60 and HLA-B27 in ankylosing spondylitis susceptibility in a Taiwanese population with contribution of the different genes to the odds ratio (OR) marked. ^1^Odds ratio is calculated by unconditional maximum likelihood estimation and 95% confidence intervals are calculated using normal approximation (Wald method). ^2^ *P*-value is calculated by Fisher-exact test. AS: ankylosing spondylitis; CI: confidence interval; No: number of individuals; OR: odds ratio.

We further assessed the independent effect and gene-gene interaction effect of *HLA-B27* and *HLA-B60* to AS susceptibility by categorizing our samples into four strata: *HLA-B27*+/*HLA-B60*+, *HLA-B27*+/*HLA-B60*-, *HLA-B27*-/*HLA-B60*+, *HLA-B27*-/*HLA-B60*-. Samples with *HLA-B27*-/*HLA-B60*- were considered as reference. As the prevalence of AS is relatively rare in Asian (0.167%), odds ratio was calculated to substitute relative risk in this study. Fig 1 showed that both *HLA-B27* and *HLA-B60* were disease-susceptibility gene for AS, with odds ratio (OR) 152 (95% confidence interval (CI) 91 to 255, Fisher’s-exact p = 0.0072) and OR 2.9 (95% CI 1.4 to 6.0, Fisher’s exact p = 1.222×10^−157^) respectively. With *HLA-B27*−/*HLA-B60*− as the reference, patient who carried both *HLA-B27* and *HLA-B60* (*HLA-B27*+/*HLA-B60*+) showed a high susceptibility to AS, with the OR increased to 201 (95%CI 85 to 475, Fisher’s exact p = 2.5007×10^−69^). Besides, CaTS power calculator revealed an expected power for a one stage study of 1.000 for both genetic risk factors to the AS susceptibility.

To confirm the independent effect of *HLA-B27*and *HLA-B60* to AS susceptibility, logistic regression analysis were performed and the result indicated that two genetic risk factors were independent for the susceptibility of AS (*HLA-B27*: *P*-value < 0.001 and *HLA-B27 P*-value = 0.0148) (Table 3).

**Table 3:** Logistic regression analysis to identify the independency of two risk factors *HLA-B27* and *HLA-B60*.

Coefficients	Estimate ß	Standard error	*P*-value
Intercept	-2.6914	0.1795	<0.001**
*HLA-B27*	4.8794	0.2326	<0.001**
*HLA-B60*	0.7493	0.3074	0.0148*

Significant (p<0.05) value is in bold* and p<0.001 is in bold**.

The risk for AS in *HLA-B27*+/*HLA-B60*+ exceeded the sum of the risks (201 > (152+2.9)) in *HLA-B27*−/*HLA-B60*+ and *HLA-B27*+/*HLA-B60*− (Fig 1) but not the product of the risks (201 < (152×2.9)). Calculated biological interaction measures show a departure from additivity of the two risk factors combined (*HLA-B27*+/*HLA-B60*+) with a RERI of 46.79 (95% CI: -117.58 to 211.16), AP of 0.23 (95% CI: -0.41 to 0.88) and S of 1.31 (95% CI: 0.56 to 3.04). These results implied the positive gene-gene interaction effects between *HLA-B27* and *HLA-B60* in a Taiwanese population.

In order to investigate the association between *HLA* antigens and AS severity, further analysis was conducted. We investigated whether *HLA-B27* or *HLA-B60* associated with clinical phenotypes including Bath Ankylosing Spondylitis Disease Activity Index (BASDAI), Bath Ankylosing Spondylitis Functional Index (BASFI), and Bath Ankylosing Spondylitis Global Index (BAS-G), which represent disease activity, physical function, and global well-being respectively. Although a deviation form additivity of *HLA-B27* and *HLA-B60* to AS susceptibility has been detected, neither association between *HLA-B27* nor *HLA-B60* and AS disease severity was observed (Table 4).

**Table 4:** Difference in the scores of BASDAI, BASFI, and BAS-G among AS patients stratified by different *HLA-B27/ HLA-B60* genotype.

	No.	BASDAI	BASFI	BAS-G
*HLA-B27* + /*HLA-B60* +	88	4.37 ± 2.20^a^	2.17 ± 2.33	4.17 ± 2.79
*HLA-B27* + /*HLA-B60* –	334	4.28 ± 2.16	2.02 ± 2.19	4.30 ± 2.73
*HLA-B27* –/*HLA-B60* +	12	4.72 ± 1.61	1.64 ± 1.40	3.65 ± 2.26
*HLA-B27* –/*HLA-B60* –	25	4.94 ± 2.58	2.60 ± 2.32	4.91 ± 3.05
P-value^b^		0.5156	0.5205	0.3995

^a^Data represent means ± S.D.

^b^P-value was calculated by Kruskal-Wallis rank sum test.

## Discussion

*HLA* has been known to involve in the antigen recognition process and is a well-known susceptibility factor for the pathogenesis of AS. *HLA-B27* is considered as the major susceptibility factor of AS [8,14,15]. *HLA-B27* is a highly polymorphic gene, with 105 subtypes: HLA-B*27:*01* to HLA-B*27:*106* [16,17]. Indeed, comparison of *HLA-B27*−/*HLA-B60*− group and *HLA-B27*+/*HLA-B60*− group (Fig 1), our results confirmed that *HLA-B27* plays an important role in the risk of AS in Taiwanese patients.

To measure biological interaction between *HLA-B27* and *HLA-B60*, we calculated three parameters that measure the departure from additivity of risk effects of each risk factor, i.e. RERI, the relative excess risk due to attributable to *HLA-B27* and *HLA-B60* interaction; AP[AB], the proportion of AS among those with both exposures that is attributable to *HLA-B27* and *HLA-B60* interaction; and synergy index, which measures the interaction between two risk factors expressed as the ratio of relative excess risk for the combined effect of the risk factors and the sum of the relative excess risks for each separate effect of the two risk factors. In our study, odds ratio was calculated to estimate the value of risk ratio, and further, calculate the value of RERI, AP and S. Our results showed a departure from additivity of the two risk factors combined (*HLA-B27*+/*HLA-B60*+) with a RERI of 46.79 (95% CI: -117.58 to 211.16), AP of 0.23 (95% CI: -0.41 to 0.88) and S of 1.31 (95% CI: 0.56 to 3.04). The RERI, AP and S indices showed a positive additive interaction, indicating that the combination effects of *HLA-B27* and *HLA-B60*.

*HLA-B27* and *HLA-B60* genes are located on chromosome 6p21.3, both coding proteins involve in antigen presenting functions. HLA presents endogenous antigens to T-cells and further triggers the autoimmune responses. Recent studies suggested that peptide motifs of *HLA-B60* may different from *HLA-B27* [18–20], thus these *HLA-B* subtypes might involve in different antigen-triggered pathologic pathways. Therefore, the epistatic effects between *HLA-B27* and *HLA-B60* may be due to the similar downstream T-cell mediated immune responses. Kirsten Falk et al. (1995) proposed that the peptide binding motif of *HLA-B60* is different from *HLA-B27* [20], thus it is unlikely that *HLA-B27* and *HLA-B60* can bind with the same AS pathogenic peptides and trigger disease onset^9^. However, López D et al. (1994) showed the cross-reactions of T cell epitope from CTL clones between *HLA-B27* with *HLA-B60/61* [21]. Thus, similar T-cell epitopes may be a key factor to explain the epistatic effects between *HLA-B27* and *HLA-B60*.

In consistence with the Floris A van Gaalen et al (2012) study, our study confirmed that *HLA-B60* can be used as an independent risk factor for AS susceptibility. In addition, *HLA-B60* showed a modest positive biological interaction effects with *HLA-B27*. However, the difference between two studies is that our results showed that the risk ratio attributed by *HLA-B27* is greater than that of *HLA-B60* suggesting that *HLA-B27* remains the most important genetic predictor on AS susceptibility in Taiwanese population. Comparatively, *HLA-B60* plays as a minor additive role. Because of the difficulties in early diagnosis of AS in Taiwan, our study revealed a possibility to the implementation of combining *HLA-B60* with *HLA-B27* screen in future clinical practice.

There are some limitations in this study. First, larger sample size is needed to confirm our finding about the interaction effect between *HLA-B27* and *HLA-B60*. Second, the mechanisms for addressing the epistatic effects between *HLA-B27* or *HLA-B60* is still unclear. Third, the interaction between genes and environment is not further investigated in this study. Despite the strong correlation between *HLA-B27* and *HLA-B60* to the AS susceptibility was found, none of significant correlation between *HLA-B27*, *HLA-B60* and clinical manifestations of AS, i.e. BASDAI, BASFI, and BAS-G [2] was observed in this study. Indeed, similar findings were observed in macrophage migration inhibitory factor (MIF) gene polymorphism. MIF is associated with the susceptibility but not severity of polyarthritis [22]. In short, *HLA-B27* and *HLA-B60* are strong genetic determinant of susceptibility to AS. Therefore, combination of these two *HLA* antigens can be applied as a better clinical tool to detect the risk of AS.

In summary, we revealed that *HLA-B60* is an independent risk factor for AS in Taiwanese population. In addition, a trend of positive interaction effects of *HLA-B27* (rs13202464) and *HLA-B60* was identified in a Taiwanese population, which is consistence with Caucasians population. Furthermore, we have shown that polymorphism between *HLA-B27* and *HLA-B60* antigens are associated with susceptibility to, but not severity of AS. As a results, Taiwanese individuals with the *HLA-B27*+/*HLA-B60*+ genotype have high risk of developing AS.
